# Perioperative Complications and In-Hospital Mortality in Paraplegic Radical Cystectomy Patients

**DOI:** 10.1245/s10434-024-16332-3

**Published:** 2024-10-08

**Authors:** Francesco Di Bello, Carolin Siech, Mario de Angelis, Natali Rodriguez Peñaranda, Zhe Tian, Jordan A. Goyal, Claudia Collà Ruvolo, Gianluigi Califano, Massimiliano Creta, Fred Saad, Shahrokh F. Shariat, Alberto Briganti, Felix K. H. Chun, Salvatore Micali, Nicola Longo, Pierre I. Karakiewicz

**Affiliations:** 1 Cancer Prognostics and Health Outcomes Unit, Division of Urology, University of Montréal Health Center, Montréal, QC Canada; 2 Department of Neurosciences, Science of Reproduction and Odontostomatology, University of Naples Federico II, Naples, Italy; 3 Department of Urology, Goethe University Frankfurt, University Hospital, Frankfurt am Main, Germany; 4 Vita-Salute San Raffaele University, Milan, Italy; 5 Division of Experimental Oncology/Unit of Urology, URI, Urological Research Institute, IRCCS San Raffaele Scientific Institute, Milan, Italy; 6 Department of Urology, Ospedale Policlinico e Nuovo Ospedale Civile S. Agostino Estense Modena, University of Modena and Reggio Emilia, Modena, Italy; 7 Department of Urology, Comprehensive Cancer Center, Medical University of Vienna, Vienna, Austria; 8 Department of Urology, Weill Cornell Medical College, New York, NY USA; 9 Department of Urology, University of Texas Southwestern Medical Center, Dallas, TX USA; 10 Hourani Center for Applied Scientific Research, Al-Ahliyya Amman University, Amman, Jordan

**Keywords:** Bladder, NIS, ICU, Surgery, Morbidity

## Abstract

**Objective:**

The aim of this study was to test for the association between paraplegia and perioperative complications as well as in-hospital mortality after radical cystectomy (RC) for non-metastatic bladder cancer.

**Methods:**

Perioperative complications and in-hospital mortality were tabulated in RC patients with or without paraplegia in the National Inpatient Sample (2000–2019).

**Results:**

Of 25,527 RC patients, 185 (0.7%) were paraplegic. Paraplegic RC patients were younger (≤70 years of age; 75 vs. 53%), more frequently female (28 vs. 19%), and more frequently harbored Charlson Comorbidity Index ≥3 (56 vs. 18%). Of paraplegic vs. non-paraplegic RC patients, 141 versus 15,112 (76 vs. 60%) experienced overall complications, 38 versus 2794 (21 vs. 11%) pulmonary complications, 36 versus 3525 (19 vs. 14%) genitourinary complications, 33 versus 3087 (18 vs. 12%) intraoperative complications, 21 versus 1035 (11 vs. 4%) infections, and 17 versus 1343 (9 vs. 5%) wound complications, while 62 versus 6267 (34 vs. 25%) received blood transfusions, 47 versus 3044 (25 vs. 12%) received critical care therapy (CCT), and intrahospital mortality was recorded in 13 versus 456 (7.0 vs. 1.8%) patients. In multivariable logistic regression models, paraplegic status independently predicted higher overall CCT use (odds ratio [OR] 2.1, *p* < 0.001) as well as fourfold higher in-hospital mortality (*p* < 0.001), higher infection rate (OR 2.5, *p* < 0.001), higher blood transfusion rate (OR 1.45, *p* = 0.009), and higher intraoperative (OR 1.56, *p* = 0.02), wound (OR 1.89, *p* = 0.01), and pulmonary (OR 1.72, *p* = 0.004) complication rates.

**Conclusion:**

Paraplegic patients contemplating RC should be counseled about fourfold higher risk of in-hospital mortality and higher rates of other untoward effects.

Radical cystectomy (RC) is guideline-recommended standard treatment in non-metastatic muscle-invasive bladder cancer.^1,2^ In rare instances, paraplegic patients may require RC.^3–5^ It is known that paraplegic patients harbored higher proportions of invasive and aggressive tumors.^6^ Moreover, it is also known that the paraplegia status may predispose to higher rates of complications and even potentially higher mortality after RC.^5–8^ Unfortunately, those data were from such small sample sizes that lack in representativity nowadays;^5–7^ however, it is unknown what the magnitude of morbidity and mortality increase is in paraplegic patients relative to non-paraplegic RC patients. To address this knowledge gap, we tested for differences in perioperative complications and in-hospital mortality between paraplegic and non-paraplegic RC patients with non-metastatic bladder cancer. Specifically, we relied on a large-scale population-based cohort of RC patients within the National Inpatient Sample (NIS, 2000–2019).

## Methods

### Data Source

To test for perioperative complications and in-hospital mortality after RC, we relied on discharge data from the NIS (2000–2019). The NIS is a set of longitudinal hospital inpatient databases included in the Healthcare Cost and Utilization Project (HCUP) and formed by the Agency for Healthcare Research and Quality (AHRQ) through a Federal-State partnership.^9^ All diagnoses and procedures were coded using the International Classification of Diseases (ICD) 9th Revision Clinical Modification (ICD-9-CM), ICD 10th Revision Clinical Modification (ICD-10-CM), and the ICD 10th Revision Procedure Coding System (ICD-10-PCS).

### Study Population

We focused on patients with a primary diagnosis of non-metastatic bladder cancer (ICD-9-CM codes 188.0–188.6, 188.8, 188.9, and ICD-10-CM codes C67.0–C67.6, C67.8, C67.9) aged ≥18 years. Only patients treated with RC were included according to previously reported methodology.^10–12^ Patients with a diagnosis of lymph node invasion or metastatic stage (ICD-9-CM codes 196.x, 197.x, 198.x, and ICD-10-CM codes C77.x, C78.x, C79.x) were excluded.^11,12^ Additionally, patients were stratified according to paraplegia status (ICD-9-CM codes 344.1, 342.x, 334.1, 342.x, 343.x, 344.0–344.6, 344.9, and ICD-10-CM codes G04.1, G11.4, G80.1, G80.2, G81.x, G82.x, G83.0–G83.4, G83.9).^13^

### Definition of Variables for Analyses

Study endpoints consisted of intraoperative and postoperative complications (infections, pulmonary, genitourinary, wound, cardiac, vascular, gastrointestinal complications and blood transfusions),^11,14^ length of stay, in-hospital mortality, and use of critical care therapies (CCTs) that consisted of invasive mechanical ventilation (IMV), total parenteral nutrition (TPN), percutaneous endoscopic gastrostomy tube, and dialysis for acute kidney failure or tracheostomy, identified by ICD-9 and ICD-10 codes according to previously established methodology.^15,16^ The Deyo modification of CCI was used to account for comorbidities as well as complications according to previous methodology relying on the NIS.^17–21^ We relied on coding algorithms for defining comorbidities in ICD-9-CM and ICD-10-CM codes reported by Quan et al.^13^ Covariates consisted of patient characteristics, including age at admission (years, continuously coded), sex (female vs. male), and Charlson Comorbidity Index (CCI, 0–2 vs. ≥3), as well as hospital characteristics, including teaching hospital status (teaching vs. non-teaching) and hospital size (large [≥400 beds] vs. medium [200–399 beds] vs. small [<200 beds]).

### Statistical Analyses

First, patient and hospital characteristics as well as perioperative length of stay, perioperative complications, and in-hospital mortality rates were tabulated. Medians and interquartile ranges (IQRs) were recorded for continuously coded variables, while frequencies and proportions were recorded for categorical variables. The Wilcoxon rank-sum test, Pearson Chi-square test, and Fisher’s exact test were applied. Second, univariable and multivariable logistic regression models (LRMs) predicting perioperative complications, overall CCT use, as well as specific CCT components, such as IMV and TPN, and in-hospital mortality were fitted after adjustment for clustering at the hospital level using generalized estimation equation methodology.^11,15^ Specifically, percutaneous endoscopic gastrostomy tube, dialysis for acute kidney failure, or tracheostomy that accounted for 1.3% of all CCT use were excluded from further analyses. All analyses and their reporting followed the NIS reporting guidelines.^9^ Due to an NIS data reporting agreement, only patient subgroups of at least 11 patients could be reported. In consequence, for patient subgroups of <11 observations, counts and associated proportions were reported as <11. All tests were two sided, with a significance level set at *p* < 0.05. R software environment was used for statistical computing and graphics (R version 4.2.2; R Foundation for Statistical Computing, Vienna, Austria).

## Results

### Descriptive Characteristics of the Study Population

Within the NIS 2000–2019, we identified 25,527 non-metastatic bladder cancer patients who underwent RC between 2000 and 2019, of whom 185 (0.7%) were paraplegic (Table 1). Paraplegic patients were younger (median age 64 vs. 70 years; *p* < 0.001), more frequently female (28 vs. 19%; *p* = 0.002), and more frequently harbored CCI ≥3 (56 vs.18%; *p* < 0.001).

**Table 1:** Descriptive characteristics of 25,527 non-metastatic bladder cancer patients undergoing radical cystectomy according to paraplegia status within the Nationwide Inpatient Sample from 2000 to 2019

		Paraplegic [*N* = 185 (0.7%)]	Non-paraplegic, [*N* = 25,342 (99.3%)]	*p*-Value^a^
Age at admission, years [median (IQR)]		64 (54, 71)	70 (62, 76)	< 0.001
Sex	Male	133 (72)	20,471 (81)	0.002
	Female	52 (28)	4871 (19)	
Charleson Comorbidity Index	≥3	104 (56)	4654 (18)	< 0.001
Teaching hospital status		184 (80)	19,720 (78)	0.5
Hospital size	Large (≥400 beds)	131 (71)	17,874 (71)	0.6
	Medium (200–399 beds)	40 (22)	4813 (19)	
	Small (<200 beds)	14 (7)	2655 (10)	

Data are expressed as *n* (%) unless otherwise specified

^a^Pearson's Chi-square test, Fisher’s exact test

*IQR* Interquartile range

### Perioperative Complications, Length of Stay, and In-Hospital Mortality Rates

Regarding perioperative complications in paraplegic versus non-paraplegic RC patients, 141 vs. 15,112 (76 vs. 60%; *p* < 0.001) experienced overall complications; 38 versus 2794 (21 vs. 11%; *p* < 0.001) experienced pulmonary complications; 36 versus 3525 (19 vs. 14%; *p* = 0.03) experienced genitourinary complications; 33 versus 3087 (18 vs. 12%; *p* < 0.001) experienced intraoperative complications; 21 versus 1035 (11 vs. 4%; *p* < 0.001) experienced infections; 17 versus 1343 (9 vs. 5%; *p* = 0.02) experienced wound complications; 62 versus 6267 (34 vs. 25%; *p* < 0.001) received blood transfusions; 47 versus 3044 (25 vs. 12%; *p* < 0.001) received CCT; 22 versus 1044 (12 vs. 4.1%) required IMV; and 26 versus 2047 (14 vs. 8.1%) required TPN after RC. Paraplegic RC patients exhibited higher length of stay (10 vs. 8 days) compared with their non-paraplegic counterparts (*p* < 0.001). In-hospital mortality was recorded in 13 versus 456 (7.0 vs. 1.8%; *p* < 0.001) patients (Table 2).

**Table 2:** Perioperative complications of 25,527 non-metastatic bladder cancer patients undergoing radical cystectomy according to paraplegia status within the Nationwide Inpatient Sample from 2000 to 2019

	Paraplegic [*N* = 185 (0.7%)]	Non-paraplegic, [*N* = 25,342 (99.3%)]	*p*-Value^a^
Overall complications	141 (76)	15,112 (60)	<0.001
Intraoperative complications	33 (18.0)	3087 (12.0)	<0.001
Postoperative complications			
Infections	21 (11.0)	1035 (4.0)	<0.001
Pulmonary complications	38 (21.0)	2794 (11.0)	<0.001
Genitourinary complications	36 (19.0)	3525 (14.0)	0.03
Wound complications	17 (9.2)	1343 (5.3)	0.02
Cardiac complications	19 (10.0)	2677 (11.0)	0.9
Vascular complications	<11 (≤5.9)	698 (2.8)	0.7
Gastrointestinal complications	54 (29.0)	6285 (25.0)	0.2
Blood transfusion	62 (34.0)	6267 (25.0)	0.006
CCT overall	47 (25.0)	3044 (12.0)	<0.001
IMV	22 (12)	1044 (4.1)	<0.001
TPN	26 (14)	2047 (8.1)	0.003
Length of stay, days [median (IQR)]	10.0 (8.0, 16.0)	8.0 (6.0, 11.0)	<0.001
In-hospital mortality	13 (7.0)	456 (1.8)	<0.001

Data are expressed as *n* (%) unless otherwise specified

^a^Wilcoxon rank-sum test, Pearson's Chi-square test, Fisher’s exact test

*CCT* critical care therapy, *IMV* invasive-mechanical ventilation, *IQR* interquartile range, *TPN* total parenteral nutrition

### Multivariable Logistic Regression Models Predicting Perioperative Complications and Mortality

In multivariable LRM (Table 3), paraplegic status independently predicted higher in-hospital mortality (odds ratio [OR] 3.97; *p* < 0.001), higher rates of infections (OR 2.57; *p* < 0.001), higher rates of IMV use (OR 2.51; *p* < 0.001), higher rates of overall CCT use (OR 2.18; *p* < 0.001), higher rates of overall complications (OR 1.99; *p* < 0.001), higher rates of wound complications (OR 1.89; *p* = 0.01), higher rates of TPN use (OR 1.75; *p* = 0.008), higher rates of pulmonary complications (OR 1.72; *p* = 0.004), intraoperative complications (OR 1.56; *p* = 0.02), blood transfusions (OR 1.45; *p* = 0.009), and longer length of stay (OR 1.31; *p* < 0.001) after RC. Conversely, no statically significant association was recorded between paraplegic status and genitourinary complications after RC (*p* = 0.07).

**Table 3:** Univariable and multivariable logistic regression models predicting the association between paraplegia status on perioperative complications, critical care therapy use, in-hospital mortality, and length of stay in 25,527 non-metastatic bladder cancer patients undergoing radical cystectomy after adjustment for clustering at the hospital level using generalized estimation equation methodology

Outcomes of interest	Univariable		Multivariable^a^	
	RR/OR (95% CI)	*p*-value	RR/OR (95% CI)	*p*-value
Overall complications	2.31 (1.60–3.32)	<0.001	1.99 (1.38–2.87)	<0.001
Intraoperative complications	1.56 (1.08–2.25)	0.02	1.56 (1.08–2.26)	0.02
Postoperative complications				
Infections	2.96 (1.89–4.65)	<0.001	2.57 (1.62–4.08)	<0.001
Wound complications	1.81 (1.10–2.97)	0.02	1.89 (1.14–3.12)	0.01
Pulmonary complications	2.02 (1.40–2.91)	<0.001	1.72 (1.19–2.49)	0.004
Genitourinary complications	1.51 (1.05–2.16)	0.02	1.39 (0.97–2.01)	0.07
Blood transfusion	1.56 (1.18–2.05)	0.001	1.45 (1.09–1.92)	0.009
CCT overall	2.40 (1.72–3.35)	<0.001	2.18 (1.55–3.08)	<0.001
IMV	3.07 (1.97–4.77)	<0.001	2.51 (1.60–3.95)	<0.001
TPN	1.78 (1.19–2.67)	0.005	1.75 (1.16–2.64)	0.008
Length of stay	1.34 (1.17–1.52)	<0.001	1.31 (1.15–1.50)	<0.001
In-hospital mortality	4.06 (2.30–7.16)	<0.001	3.97 (2.19–7.19)	<0.001

^a^Adjusted for age at admission, sex, and Charlson Comorbidity Index

*CCT* critical care therapy, *CI* confidence interval, *IMV* invasive-mechanical ventilation, *RR* rate ratio, *OR* odds ratio, *TPN* total parenteral nutrition

## Discussion

In non-metastatic bladder cancer patients undergoing RC, the magnitude of morbidity and mortality increase in paraplegic patients relative to non-paraplegic RC patients is unknown. To address this knowledge gap, we relied on a large-scale population-based cohort within the NIS (2000–2019) and made several noteworthy observations.

First, we identified important differences in the descriptive characteristics between RC patients with and without paraplegia. Specifically, paraplegic RC patients were younger (64 vs. 70 years of age; *p* < 0.001) and sicker (CCI ≥3: 56 vs. 18%; *p* < 0.001). Unexpectedly, a higher proportion of paraplegic patients were female (28 vs. 19%; *p* = 0.002). Based on these differences, analyses addressing adverse intraoperative and hospital outcomes, such as the current analyses, require detailed multivariable adjustment for baseline patient variables, as was done in the present study.

Second, we postulated that adverse in-hospital outcomes will be significantly higher in paraplegic versus non-paraplegic RC patients. The most important adverse outcome consists of in-hospital mortality. Testing for in-hospital mortality rates revealed that paraplegic RC patients had fourfold higher mortality than their non-paraplegic counterparts (7.0 vs. 1.8%; *p* < 0.001; multivariable OR 3.97; *p* < 0.001). These observations emphasized the need for preoperative counseling in paraplegic RC candidates. These individuals should be aware that the risk of in-hospital death is significantly more frequent than their non-paraplegic counterparts. In consequence, paraplegic patients should be optimized to the best possible extent prior to RC. Preoperative optimization in such patients is even more important than in their non-paraplegic counterparts due to the recorded fourfold higher in-hospital mortality risk. Moreover, it is of utmost importance to improve the screening of this population as a high priority. It is well known that paraplegic patients, due to their method of bladder emptying, are sharply more exposed to bladder cancer incidence at an advanced stage.^6,22^ In consequence, follow-up and screening approaches must therefore be intensified as long as the paraplegic status exists.

Third, we also addressed rates of intra- and postoperative complications. Unlike the fourfold increase in in-hospital mortality, the rates of excess complications were not as pronounced; specifically, they ranged from 1.4 to 2.5. The three complication categories with the highest increase in paraplegic patients were infections (OR 2.57; *p* < 0.001), wound (OR 1.89; *p* = 0.01) and pulmonary (OR 1.72; *p* = 0.004). The potential explanation of these negative results may be related to prolonged bed-ridden status, poor mobility, and the more difficult cleaning for paraplegic RC patients that invariably has worsened by the paraplegic status itself. In consequence, paraplegic patients should be counseled preoperatively about these three possibly higher specific complication categories.

Fourth, we also examined overall CCT rates as well as specific CCT components, such as IMV and TPN. Paraplegic patients exhibited a twofold higher rate of overall CCT, relative to their non-paraplegic counterparts. Specifically, these patients exhibited a substantially (2.5-fold) higher risk of IMV as well as a (1.7-fold) higher risk of TPN. The paraplegic RC candidates should be counseled preoperatively about the twofold higher CCT risk. Of CCT components, IMV and TPN were most frequent in that order. The remaining CCT modalities that included percutaneous endoscopic gastrostomy tube, dialysis for acute kidney failure, or tracheostomy accounted for 1.3% of all CCT use. In consequence, optimization of the cardiorespiratory status as well as gastrointestinal function should be invariably included in preoperative assessment of paraplegic RC candidates.

Taken together, although paraplegic RC patients were younger, they exhibited higher comorbidity burden (CCI ≥3). Moreover, paraplegic RC patients exhibited a fourfold higher in-hospital mortality rate. They also exhibited 1.4- to 2.5-fold higher rates of various complications, as well as twofold higher overall CCT use that predominantly consisted of IMV and TPN. These increases in risk should be addressed at preoperative counseling and patient optimization. Moreover, it is of utmost importance to improve the screening of this population as a high priority. It is well known that paraplegic patients, due to their method of bladder emptying, are sharply more exposed to bladder cancer incidence at an advanced stage.^6,22^ In consequence, follow-up and screening approaches must therefore be intensified as long as the paraplegic status exists.

Despite the novelty of our observations, the present study has inherent limitations. First, it was a retrospective study with selection and reporting biases, as in previous NIS^14,15,20,23^ or Surveillance Epidemiology, and End Results studies.^24–26^ Second, the counts of paraplegic patients are relatively small due to their rarity. Despite this aspect, the current population-based study includes a larger number of paraplegic patients than those analyzed in single-institution repositories.^5,6,22^ Third, details of RC, such as timing and/or duration of surgery or type of urinary diversion performed after RC, are unavailable. Fourth, surgical and/or institutional expertise cannot be accounted for. Finally, within the NIS, only in-hospital data are available. Nevertheless, the present study provides important and new insights into the perioperative risks of paraplegic RC patients.

## Conclusion

Paraplegic patients contemplating RC should be counseled about the fourfold higher risk of in-hospital mortality and higher rates of other untoward effects. Moreover, screening of paraplegic patients should gain importance in order to prevent the advent of adverse in-hospital outcomes when RC becomes mandatory. In consequence, physicians must provide stricter follow-up, and screening approaches must therefore be intensified as long as the paraplegic status occurs.

## Data Availability

All analyses and their reporting followed the NIS reporting guidelines. The specific datasets generated and/or analyzed during the current study are available from the corresponding author on reasonable request.
